# A Yet Unrecognized Cause of Unusually High Levothyroxine Replacement Dose: Protein-Losing Enteropathy

**DOI:** 10.1016/j.aace.2023.04.004

**Published:** 2023-04-13

**Authors:** Run Yu

**Affiliations:** Division of Endocrinology, University of California Los Angeles David Geffen School of Medicine, Los Angeles, California

**Keywords:** levothyroxine dose, hypoalbuminemia, protein-losing enteropathy, protein wasting

## Abstract

**Background/Objective:**

Large amount of protein wasting such as in nephrotic syndrome is a rare cause of high levothyroxine (LT4) replacement dose requirement. A case has been reported here that demonstrates that protein-losing enteropathy is a novel and yet unrecognized cause of high LT4 replacement dose requirement.

**Case Report:**

A 21-year-old man with congenital heart disease was found to have primary hypothyroidism and started LT4 replacement. His weight was approximately 60 kg. Nine months later, while he was taking LT4 100 μg daily, thyroid-stimulating hormone (TSH) level was >200 μIU/mL (normal range, 0.3-4.7 μIU/mL) and free thyroxine level was 0.3 ng/dL (normal range, 0.8-1.7 ng/dL). The patient had excellent medication compliance. LT4 dose was increased to 200 μg daily and then 200 and 300 μg every other day. Two months later, TSH level was 3.1 μIU/mL and free thyroxine level was 1.1 ng/dL. He did not exhibit malabsorption or proteinuria. His albumin levels had been low since the age of 18 years (mostly <2.5 g/dL). Stool α-1-antitrypsin levels and calprotectin levels were elevated on multiple occasions. Protein-losing enteropathy was diagnosed.

**Discussion:**

As most circulating LT4 is protein-bound, loss of protein-bound LT4 due to protein-losing enteropathy is the most plausible cause of the large LT4 dose requirement in this case.

**Conclusion:**

This case demonstrates that protein-losing enteropathy, through loss of protein-bound thyroxine, is a novel and yet unrecognized cause of high LT4 replacement dose requirement. In patients who require high LT4 dose for unclear reasons, albumin levels should be examined and protein wasting be suspected in those with low albumin levels.

**Highlights**

• Protein wasting results in loss of protein-bound thyroxine
• Protein wasting needs to be considered in those requiring high levothyroxine dose
• Protein-losing enteropathy is a novel cause of high levothyroxine dose
• Hypoalbuminemia is a clue to protein-losing enteropathy

**Clinical Relevance**

Protein-losing enteropathy, through loss of protein-bound thyroxine, is a novel and yet unrecognized cause of high L4 replacement dose requirement. In patients who require high LT4 dose for unclear reasons, albumin levels should be examined and protein wasting be suspected in those with low albumin levels.

## Introduction

In patients with overt hypothyroidism, an adequate daily oral levothyroxine (LT4) replacement dose is usually approximately 1.6 μg/kg body weight.^1^ Not infrequently, some patients apparently require higher doses than what their body weight would predict, commonly due to poor medication compliance and decreased LT4 absorption.^2^^,^^3^ As most natural or synthetic thyroxine in circulation is protein-bound, a large amount of protein wasting such as in nephrotic syndrome is a rare cause of high LT4 replacement dose requirement.^4^ A case has been reported here that demonstrates that protein-losing enteropathy, through loss of protein-bound thyroxine, is a novel and yet unreported cause of high LT4 dose requirement.

## Case Report

A 21-year-old man was seen by the inpatient endocrine consult service for hypothyroidism. He had been found at birth to have congenital heart disease. At 4 years of age, he received orthotopic heart transplantation. At the age of 21 years, he underwent a second orthotopic heart transplantation. His thyroid function had been normal (thyroid-stimulating hormone [TSH] level, 1.8-3.6 μIU/mL) from age 9 to 19 years. Six weeks after the second heart transplant, while still in the intensive care unit for renal insufficiency and recurrent ascites and pleural effusion, he developed cold intolerance, fatigue, and depressed mood; thyroid function was tested and TSH level was 10.6 μIU/mL (normal range, 0.3-4.7 μIU/mL), free thyroxine (T4) 0.6 ng/dL (normal range, 0.8-1.7 ng/dL), and free triiodothyronine 80 pg/dL (normal range, 222-383 pg/dL) (Table); thyroid peroxidase antibody level was normal. He had been taking pantoprazole 40 mg daily for 2 months for gastric reflux. There was a history of hypothyroidism in his maternal grandmother and possible hypothyroidism in his mother. His weight was 57.1 kg. No goiter or thyroid nodules were found by physical examination. He was diagnosed with primary hypothyroidism with likely coexisting euthyroid sick syndrome and started intravenous LT4 50 μg daily due to ongoing tube feeding. Nine days later, LT4 was switched to oral administration at various doses (Table). At the time of discharge after a lengthy inpatient stay of 9 months (2.5 months after starting LT4), LT4 dose was 100 μg every Sunday and 88 μg on all other days of the week, and TSH level was 14.3 μIU/mL and free T4 level was 0.5 ng/dL; LT4 dose was increased to 100 μg orally daily. The patient missed an endocrine outpatient appointment due to intercurrent end-stage renal disease and hemodialysis. Six months later, he was admitted for sepsis. Before discharge, although the patient had largely recovered from sepsis, TSH level was >200 μIU/mL and free T4 level was 0.3 ng/dL. The inpatient endocrine service was consulted again. The patient maintained that he regularly took LT4 100 μg every morning while fasting and did not take any other medications or supplements with it and waited for at least 1 hour before eating breakfast and taking other medications or supplements, which was corroborated by his mother who had been very closely involved in his care. He felt cold and fatigued. His weight was 62.5 kg. LT4 dose was increased to 200 μg orally daily. Two months later, TSH level was 33.35 μIU/mL and free T4 level was 1.2 ng/dL. LT4 dose was further increased to 200 μg alternating with 300 μg every other day. Another 2 months later, TSH level was 3.1 μIU/mL and free T4 level was 1.1 ng/dL (Table).

**Table:** Thyroid Function Test Results and Thyroid Replacement Over Time

Timeline	TSH, μIU/mL (normal range, 0.3-4.7)	Free thyroxine, ng/dL (normal range, 0.8-1.7)	Free triiodothyronine, pg/dL (normal range, 222-383)	Levothyroxine, μg daily
Age 9-19 y	1.8-3.6			
Age 21 y, at presentation	10.6	0.6	80	None Started 50 intravenously
9 d after presentation	4.1	0.7		Increased to 88 orally
16 d after presentation	0.54			88→75 orally
5 wk after presentation	0.39	0.3	86	176 for 2 d, then 88 orally
1.5 mo after presentation	10.9	0.8		100 orally × 1 d and 88 × 6 d per week
2 mo after presentation	14.3	0.5		88→100 orally
8 mo after presentation	>200	0.3		100→200 orally
10 mo after presentation	33.35	1.3		200→average 250 orally
12 mo after presentation	3.1	1.1		Average 250 orally
1-3 y after presentation	0.29-12.72	1.1-1.2		Average 250 orally

Abbreviation: TSH = thyroid-stimulating hormone.

Malabsorption of oral LT4 was initially suspected. The patient was thin all his life. He had chronic intermittent nausea and vomiting due to congenital foregut malrotation and gastric reflux. He did not have diarrhea and was not edematous. The patient, however, did not exhibit malabsorption of other medications or supplements. His 25-hydroxyvitamin D levels were 28 to 42 ng/mL (normal range, 20-50 ng/mL), while he took only 1000 units of cholecalciferol daily. Stool pancreatic elastase-1, stool neutral fats (monoglycerides, diglycerides, and triglycerides), and stool split fats (free fatty acids) were all normal, indicating normal pancreatic enzyme secretion and nutrient absorption. His albumin levels had been low since the age of 18 years except for a brief period when he received intravenous albumin infusion (Figure). The hypoalbuminemia was not addressed in depth before the second heart transplant as it was considered a low priority. Albumin levels after initiation of LT4 treatment were mostly <2.5 g/dL (normal range, 3.9-5.0 g/dL). Total protein was also low concurrently (Figure). Between 18 and 20 years, his renal function was normal with negative urine protein. Protein-losing enteropathy was suspected. Stool calprotectin levels were 70, 55, and 82 μg/g (≤49) on multiple measurements and α-1-antitrypsin 0.95 mg/g (<0.50); 24-hour stool α-1-antitrypsin was measured twice, and the levels were 185 and 295 mg/dL (<55), respectively. Colonoscopy (with biopsy) results were unremarkable, and esophagogastroduodenoscopy demonstrated normal esophagus and largely normal gastric mucosa except for mild antral erythema. White pinpoint villi and a chain of nodular mucosa with dilated lymphatics consistent with lymphangiectasia were found in the second portion of the duodenum. Gastric biopsy showed mild chronic gastritis with focal minimal activity, and duodenal mucosa biopsy revealed lymphangiectasia and prominent eosinophilia. Protein-losing enteropathy due to plausible eosinophilic gastroenteritis was diagnosed. The high LT4 dose requirement was attributed to gastrointestinal loss of protein-bound thyroxine, analogous to renal loss of protein-bound thyroxine in nephrotic syndrome.

**Fig:**
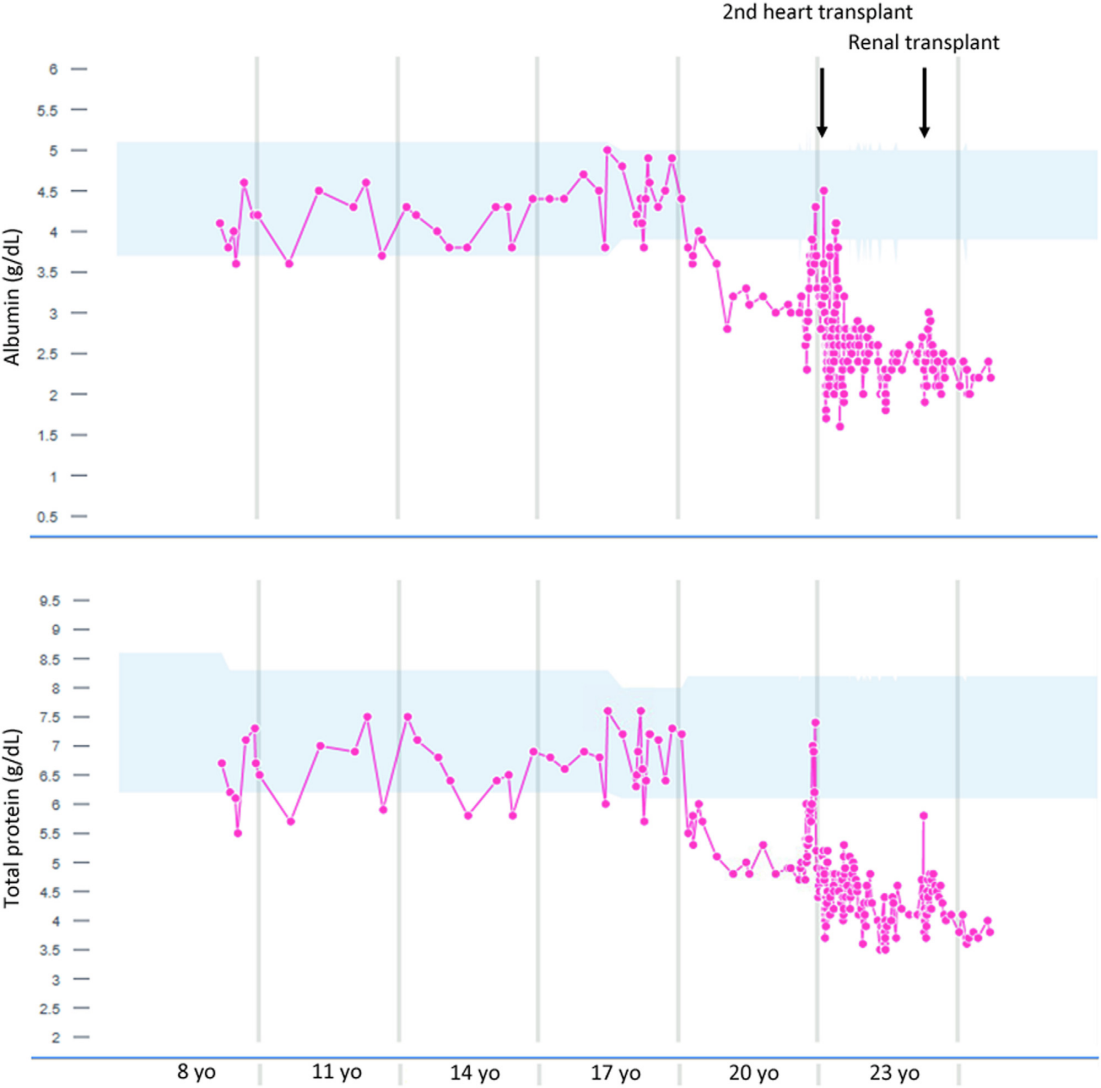
Serum albumin and total protein levels over time. Pink, albumin or total protein levels. Blue, normal range of albumin or total protein. He received albumin infusion around the second heart transplant and intravenous immunoglobulin (IVIG) infusion around the second heart transplant and the renal transplant. Note the persistent and severe hypoalbuminemia and hypoproteinemia since the age of 18 years other than when he received infusion of albumin or IVIG.

Despite a high protein diet, his albumin and total protein levels remained low (Figure). He continued the average LT4 dose of 250 μg daily in the next 2 years, and TSH levels remained largely normal (range, 0.31-3.6 μIU/mL) with occasional fluctuations (range, 0.29-12.72 μIU/mL) and free T4 levels of 1.1 to 1.2 ng/dL. His weight remained stable. He underwent renal transplant 2 years after presentation, which normalized renal function; his urine protein examination results remained negative.

## Discussion

The patient described here has primary hypothyroidism based on elevated TSH and low free T4 and triiodothyronine levels on most occasions, except when he likely had euthyroid sick syndrome in the few weeks right after he was diagnosed with hypothyroidism. His weight-based estimate of LT4 dose requirement is approximately 100 μg daily; he, however, requires an average dose of 250 μg to normalize TSH. His large LT4 replacement dose requirement is unusual and eventually attributed to loss of protein-bound thyroxine due to protein-losing enteropathy.

Poor medication compliance is a common cause of high LT4 replacement dose.^2^ In one study, about 17% of patients requiring an average of 248 μg of LT4 admit poor compliance.^2^ This patient has an excellent record of medication compliance so that poor medication compliance is unlikely the cause of his large LT4 dose requirement. Moreover, the usual LT4 dose administered by hospital staff while he was admitted also did not normalize TSH, further supporting that the high LT4 dose requirement is not related to medication compliance in this patient. Another possible factor that may contribute to his large LT4 dose requirement is the use of proton pump inhibitor. Although often cited as a cause of large LT4 dose requirement, use of proton pump inhibitor only has a marginal effect on LT4 absorption; only approximately 20% of patients on LT4 treatment receiving new proton pump inhibitor therapy require dose increase, and the average dose increase is only approximately 35%.^5^^,^^6^ Thus, the use of proton pump inhibitor does not likely explain his very large LT4 dose requirement (150% higher than weight-based estimate). LT4 malabsorption due to intrinsic gastrointestinal diseases is another concern in this patient. Gastritis, gastroparesis, gastric bypass surgery, celiac disease, lactose intolerance, and exocrine pancreatic deficiency are all known to reduce LT4 absorption.^7^ In this patient, there is no evidence of any of the above known gastrointestinal causes of LT4 malabsorption. Malabsorption can be seen in eosinophilic gastroenteritis but is rare and associated with severe symptoms and bowel obstruction.^8^ The absence of these symptoms in this patient suggests that his eosinophilic gastroenteritis unlikely causes malabsorption. His normal fecal fat, unimpaired absorption of other medications such as vitamin D and immunosuppressants, and normal iron and B12 levels also make LT4 malabsorption unlikely. The low serum albumin and globulin levels eventually led to the diagnosis of protein-losing enteropathy, which is the most plausible cause of the large LT4 dose requirement. He clearly does not have nephrotic syndrome given the absence of proteinuria.

The vast majority of circulating thyroid hormone molecules are bound to 3 plasma proteins, thyroxine-binding globulin, transthyretin, and albumin.^9^ General loss of plasma proteins, including the 3 thyroxine-binding proteins, such as by the nephrotic syndrome, results in loss of protein-bound thyroxine and increased LT4 dose requirement.^10^^,^^11^ The importance of circulating thyroid hormone binding proteins in maintaining circulating thyroid hormone levels is also illustrated in the effective reduction of free thyroxine levels by plasmapheresis for thyroid storm treatment.^12^ Protein-losing enteropathy is a syndrome of loss of circulating proteins to the gut lumen.^13^^,^^14^ It is rational to hypothesize that the loss of protein-bound thyroxine in protein-losing enteropathy greatly increases the requirement of LT4 replacement dose, as in nephrotic syndrome. Although only protein-bound thyroxine is lost, free T4 levels are reduced and TSH appropriately increased in nephrotic syndrome.^11^ These findings were also demonstrated in this case. The mechanisms responsible for reduced circulating free thyroxine levels and tissue thyroxine delivery by loss of circulating thyroid hormone binding proteins appear to be complex; reduced thyroxine reserve in circulation and decreased thyroxine levels in extravascular space may both play a role.^15^ The cause of this patient’s protein-loss enteropathy is plausibly eosinophilic gastroenteritis.^16^^,^^17^

## Conclusion

This case demonstrates that protein-losing enteropathy, through loss of protein-bound thyroxine, is a novel and yet unrecognized cause of high LT4 replacement dose requirement. In patients who require high LT4 dose for unclear reasons, albumin levels should be examined and protein wasting be suspected in those with low albumin levels.

## Disclosure

The authors have no multiplicity of interest to disclose.
